# Dental Management Considerations for Patients with Cardiovascular Disease—A Narrative Review

**DOI:** 10.31083/j.rcm2308261

**Published:** 2022-07-20

**Authors:** Kanishk Gupta, Santhosh Kumar, Meena Anand Kukkamalla, Vani Taneja, Gufaran Ali Syed, Fawaz Pullishery, Mohammad A Zarbah, Saeed M. Alqahtani, Mohammed A. Alobaoid, Saurabh Chaturvedi

**Affiliations:** ^1^Department of Periodontology, Dentistry Program, Batterjee Medical College, 21442 Jeddah, Saudi Arabia; ^2^Department of Periodontology, Manipal College of Dental Sciences, Manipal, Manipal Academy of Higher Education, 576104 Manipal, Karnataka, India; ^3^Department of Periodontology, Faculty of Dentistry, Manipal University College Malaysia (MUCM), 75150 Melaka, Malaysia; ^4^Department of Pediatric Dentistry, Dentistry Program, Batterjee Medical College, 21442 Jeddah, Saudi Arabia; ^5^Department of Endodontics, Dentistry Program, Batterjee Medical College, 21442 Jeddah, Saudi Arabia; ^6^Division of Dental Public Health, Dentistry Program, Batterjee Medical College, 21442 Jeddah, Saudi Arabia; ^7^Department of Prosthetic Dental Science, College of Dentistry, King Khalid University, 62529 Abha, Saudi Arabia; ^8^Department of Prosthetic Dentistry, College of Dentistry, King Khalid University, 62529 Abha, Saudi Arabia; ^9^Department of Restorative Dental Sciences and Department of Dental Education, King Khalid University College of Dentistry, 61471 Abha, Saudi Arabia; ^10^Department of Prosthetic Dental Science, College of Dentistry, King Khalid University, 62529 Abha, Saudi Arabia

**Keywords:** anticoagulants, cardiac arrhythmias, dental care, hypertension, myocardial ischemia, vasoconstrictor

## Abstract

Dental therapists encounter patients with various systemic diseases of which cardiovascular disease (CVD) patients form a significant segment. Relation between oral health and cardiac diseases has been well established. Common cardiac disorders encountered in a dental practice include arterial hypertension, heart failure, ischemic heart disease, cardiac arrhythmias, infective endocarditis, stroke, and cardiac pacemaker. Patients with CVDs pose a significant challenge to dental therapy. These patients need special considerations and an adequate understanding of the underlying cardiovascular condition to provide safe and effective dental treatment. Based on the cardiac condition, an appropriate modification in dental care is crucial. A multidisciplinary approach including the patient’s cardiologist can potentially reduce complications and improve dental treatment results. This review aims at unfolding the risks associated with the dental management of a cardiac patient and outlines the measures to be undertaken for optimum dental treatment.

## 1. Introduction 

Assessing a patient’s medical history is the first step of any dental treatment. A compromised medical status can alter the dental treatment plan and, if ignored, can lead to severe and sometimes fatal consequences. Cardiovascular diseases are the leading global cause of death [1, 2, 3]. In 2019, an estimated 17.9 million deaths occurred due to cardiovascular diseases constituting 32% of all the deaths worldwide [2]. With extensive improvement in healthcare facilities and an increase in life expectancy, dentists are encountering more and more elderly and medically compromised patients. In a dental practice, though syncope is the most common medical emergency reported [4], cardiovascular events are not very infrequent [5, 6]. Thus, it is very critical for dental practitioners to possess adequate knowledge, skills, and resources to counter such emergencies effectively. However, since a patient’s medical status plays a pivotal role in formulating the treatment plan, a dentist should not hesitate to contact the patient’s physician to enquire about the medical details and discuss the dental treatment plan.

This article discusses the potential cardiovascular problems a dental practitioner encounters and dentists’ management strategies in a dental clinic.

## 2. Hypertension

Arterial hypertension is one of the most common chronic medical conditions affecting the population and a significant cause of death globally. Around 10.4 million people die every year due to raised blood pressure [7]. More than 200 million people in India suffer from hypertension [8]. Hypertension can be classified as primary hypertension and secondary hypertension. Primary or ‘essential’ hypertension does not have an apparent underlying cause, while secondary hypertension has specific reasons like hyperthyroidism, vascular diseases, and adrenal medullary dysfunction [9]. 90–95% of the patients with high blood pressure in the United States fall into ‘Essential hypertension’ category [10]. Generally, hypertension is diagnosed based on an average of two or more elevated measurements taken on two or more instances [11, 12].

In 2018, *the European Society of Cardiology/European Society of Hypertension (ESC/ESH)* updated the arterial hypertension management guidelines and included blood pressure classification for all ages from 16 years [13]. According to this classification, hypertension is described as office systolic BP ≥140 mm Hg and/or diastolic BP ≥90 mm Hg. This categorization is different from the one recommended in the 2017 *ACC/AHA High Blood Pressure Clinical Practice Guidelines*, which defined hypertension as systolic blood pressure (SBP) ≥130 mm Hg or a DBP ≥80 mm Hg [14].

Hypertension can be treated by non-pharmacological or pharmacological therapy. Non-pharmacological means include diet modification, regular exercise, weight control, smoking cessation, and limiting alcohol, salt, and caffeine intake [15, 16]. Pharmacologically, antihypertensive drugs are given as monotherapy or combination therapy to control blood pressure [17]. Despite the advancement in diagnostic and therapeutic modalities, most cases of hypertension are either undiagnosed or uncontrolled. Such cases pose a significant public health problem for developing and developed nations. With increased blood pressure, the risk of myocardial infarction, heart failure, stroke, and kidney disease increases significantly.

Undetected or insufficiently controlled hypertension can be a reason for complications in a dental setup. Therefore, a thorough evaluation of a hypertensive patient visiting a dental clinic is imperative for the delivery of efficient and safe dental care. In addition, dentists can play a pivotal role in detecting and timely referring hypertensive patients to medical practitioners. For this, oral health care providers should be well versed with the recent guidelines, measurement, diagnosis, and management of hypertensive dental patients.

Before undertaking any dental procedure, it is recommended to do a patient’s complete risk assessment. Proper patient evaluation with detailed medical and family history will help assess the chances of any adverse cardiovascular event occurring during the dental treatment. In a dental office, vital signs including pulse and blood pressure should be measured for all the patients at every visit [18]. In addition, blood pressure monitoring for hypertensive patients with systemic complications must be carried out even during the procedure [19].

According to the American Dental Association, the guidelines for outpatient dental care for adult hypertensive patients are as follows [20]:

• Hypertensive patients with blood pressure less than 160/100 mmHg need no elective or emergency treatment modification.

• For patients with >160/100 mmHg of blood pressure, B.P. measurements must be repeated.

• If B.P. is reduced or is within the permitted limit according to the physician, then the emergency or elective dental care can be continued.

• However, if B.P. is confirmed to be more than 160/100 mmHg, then no elective dental treatment should be provided, and the patient should be referred to a physician.

• Emergency care can be initiated if SBP is 160–180 mmHg and/or diastolic pressure 100–109 mmHg, and blood pressure must be measured every 10–15 minutes during the procedure.

• If confirmed SBP >180 and/or DBP >109, the dentist should consult the physician before any procedure.

• Patients with SBP >180 mmHg and/or DBP >110 mmHg must undergo physician consultation immediately, or the patient must be sent for urgent medical evaluation if symptomatic.

Short morning appointments are preferable with adequate stress and anxiety management. Anxiolytic agents like diazepam (5–10 mg) the night before and one hour before the dental appointment may be helpful. Sudden positional changes during or at the end of the dental procedure should be avoided, especially in older patients to prevent orthostatic hypotension. Pain during a dental procedure can cause endogenous catecholamine release, leading to undesirable hemodynamic alterations in cardiac patients [21]. To prevent this complication, pain is controlled by using local anesthetics with vasoconstrictors like epinephrine which prolong the anesthetic effect and improve hemostasis. However, epinephrine due to its non-selective adrenergic profile causes an increase in heart rate (HR) and blood pressure (BP) making its use controversial in cardiac patients. Nevertheless, 1 or 2 cartridges of local anesthetic with 1:80,000, 1:100,000, or 1:200,000 of epinephrine are considered safe in patients with controlled hypertension and/or coronary disease [22]. Thus, a limited dose of vasoconstrictor (0.018 to 0.036 mg) [23], proper aspiration, and slow injection technique can prevent systemic absorption of vasoconstrictor and prevent cardiovascular stimulation in hypertensive patients [24].

Excessive intraoperative bleeding in hypertensive patients may be of concern, especially during an extensive surgical procedure or in patients taking anticoagulants like aspirin or warfarin. Therefore, assessing proper coagulation and limiting the surgical area should be considered [15]. Dental practitioners should be well acquainted with antihypertensive drugs used commonly like alpha-blockers, beta-blockers, diuretics, angiotensin-converting enzyme (ACE) inhibitors, and calcium channel blockers. Knowledge of oral adverse effects [15, 25] of these drugs like xerostomia, gingival hyperplasia, lichenoid reaction, orthostatic hypotension, gingival bleeding, and loss of taste aids in better patient management.

Drug-drug interactions may be encountered with antihypertensive drugs and drugs commonly prescribed by dental professionals such as NSAIDs (causing decreased antihypertensive effect) [15, 26], epinephrine (causing a transient increase in BP) [15, 26], local anesthetics (causing decreased rate of amide metabolism) [15], sedatives (causing increased sedation) [15], systemic antifungals, and opioids. Understanding drug interactions and managing the side effects of these drugs can lead to a more holistic and effective dental management of hypertensive patients [15].

### 2.1 Hypertensive Crisis

According to the 2003 Joint National Committee (JNC-7) report, a significant increase in systolic blood pressure (SBP >180 mmHg) or diastolic blood pressure (DBP >120 mmHg) is described as a ‘Hypertensive Crisis’ [27]. It is further divided into *hypertensive urgency* (situations without progressive target organ dysfunction) and *hypertensive emergency* (situations with impending or progressive target organ dysfunction) [27]. About 1–2% of hypertensive patients can ultimately develop a hypertensive crisis [28]. A dental patient manifesting the symptoms of hypertensive emergency must be immediately admitted to the intensive care unit for rapid blood pressure control using parenteral antihypertensives to prevent or limit the target organ damage [27, 28]. In case of hypertensive urgency, low-dose oral antihypertensives are used to decrease the BP gradually over hours to days.

### 2.2 Whitecoat Hypertension (WCH) and Whitecoat Effect (WCE)

*Whitecoat hypertension* can affect 15–30% of the people with more prevalence in the older population [29]. WCH describes persistently elevated office blood pressure with normal out-of-office BP in untreated patients [30]. The whitecoat effect is different from white coat hypertension. A transient increase in blood pressure in a medical environment due to the alerting response to a doctor or nurse is *whitecoat effect * [31, 32]. It can occur in both hypertensive patients and normotensive people.

The risk of cardiovascular events and hypertension-mediated organ damage (HMOD) is less associated with white coat hypertension. Still, it can be related to increased chances of long-term cardiovascular problems, as reported by recent studies [33, 34]. Therefore, patients with whitecoat hypertension are encouraged to make necessary lifestyle modifications to reduce cardiovascular risk and undergo regular follow-up appointments with consistent out-of-office blood pressure monitoring. In addition, antihypertensive drug treatment can be considered for the whitecoat hypertensive patients with a high cardiovascular risk profile [13].

## 3. Ischemic Heart Disease

Ischemic heart disease (IHD) or coronary heart disease (CHD) is a pathological condition characterized by a discrepancy between myocardial oxygen demand and supply due to reduced cardiac blood flow [35]. Blockage of coronary arteries due to the atherosclerotic plaque is the most common cause of IHD. Other causes include coronary artery spasm, coronary arteritis, embolism or shock secondary to hypotension, etc. Due to its pathophysiology, IHD is also known as coronary artery disease (CAD).

According to WHO Global Health Estimates, IHD is the leading cause of mortality globally, causing 16% of deaths alone [36]. In addition, more than half of all the cardiovascular events in patients less than 75 years of age are due to IHD. After the age of 40, the risk for developing the IHD is 49% in males and 32% in females [37]. IHD can be categorized as chronic coronary syndromes or acute coronary syndromes. Acute coronary syndromes (ACS) include unstable angina pectoris and acute myocardial infarction. Stable angina is one of the types of Chronic Coronary Syndromes (CCS).

Dental practitioners must be well aware of the significant risk factors associated with IHD and their level of control to estimate the likelihood of the dental procedure concluding uneventfully. Some non-modifiable risk factors for IHD are age, male gender, family history, and co-morbidities like kidney disease, thyroid disease, and type I and II diabetes along with others. However, the most common and modifiable risk factors include lipid disorders, high blood pressure, smoking, alcohol, diabetes, obesity, and stress [38]. In the early stages, the disease is generally asymptomatic. Nevertheless, as atherosclerosis worsens, symptoms appear, especially during emotional or physical stress when oxygen demand increases. Symptoms may include fatigue, shortness of breath, chest pain (angina), and ankle edema.

*Angina pectoris *is a sudden onset, substernal pain in the chest due to myocardial ischemia without infarction or necrosis. The pain may radiate to the left arm, left jaw, neck, teeth, or back. Anginal pain radiating to the mandible can mimic the pain of dental origin. Also, the probability of an anginal attack in a dental environment rises due to anxiety, fear, and pain [39]. Thus, a dental practitioner must be well aware of the clinical manifestations of angina and its management in the dental clinic.

Angina is categorized as stable, unstable, or Prinzmetal angina.

• Stable angina is precipitated by physical exertion or emotional stress and lasts for less than 15 minutes.

• Unstable angina represents a more severe and longer-lasting form of angina that occurs at rest without evidence of necrosis.

• Prinzmetal/variant angina occurs at rest and is most likely due to coronary artery spasm.

Complete blockage of one or more coronary arteries leads to persistent ischemia and irreversible necrosis of myocardial tissues, which eventually lead to heart attack or myocardial infarction.

Diagnosis of IHD includes assessment of symptoms, resting electrocardiogram (ECG), stress ECG, coronary computed tomography angiography (CTA), stress cardiac magnetic resonance (CMR) perfusion imaging, positron emission tomography (PET), or single-photon emission computed tomography (SPECT) perfusion imaging. In addition, invasive coronary angiography (ICA) is recommended to diagnose and manage patients with a high likelihood of IHD.

Treatment of IHD comprises the following:

(1) Antithrombotic/antiplatelet therapy- low dose aspirin [40, 41], clopidogrel [42], ticagrelor [43];

(2) Antianginal medication- sublingual nitroglycerin;

(3) Modification of cardiovascular risk factors [44] — reducing low-density lipoproteins (LDL), healthy diet, smoking cessation, and exercise;

(4) Revascularization- percutaneous coronary intervention (PCI) or coronary artery bypass grafting (CABG).

There is a high risk of recurrence of cardiovascular events in patients with myocardial infarction (MI). Previous AHA guidelines recommended that any dental surgery within six months of MI should be avoided due to the high risk of complications during this duration [45]. The emerging evidence in cardiovascular disease management advocates that no elective dental care should be given within 30 days of MI. Any emergency care during this period should be given after the physician’s consultation and in a hospital-based setup. After one month, if the patient is symptom-free, elective dental care can be provided with caution [18].

Patients with angina or a history of myocardial infarction are managed similarly in a dental setup. Though patients with unstable angina must be immediately referred to a physician, stable angina patients can be safely provided with elective dental care. Short morning appointments are preferred. This view has been rebutted, and early morning or late afternoon appointments are advised to be avoided in IHD patients [46]. Fear, pain, anxiety, and stress can precipitate angina and must be kept to a minimum during the dental visit of an IHD patient. Administration of oral anxiolytics, nitrous oxide or intravenous sedation, and maintaining profound local anesthesia with long-acting anesthetics like bupivacaine minimize the release of endogenous catecholamines. Vasoconstrictors should be used sparingly as their absorption can increase the heart rate. Using 1:100,000 or lesser concentrations of vasoconstrictor and avoiding intravascular injection with proper aspiration are advisable. It is recommended not to use more than one (2 mL) or two cartridges (4 mL) of 2% lidocaine with 1:100,000 epinephrine (0.018 to 0.036 mg of adrenaline) for dental procedures in patients with cardiovascular disease [23, 47].

If an IHD patient develops chest pain during the dental procedure and angina is suspected, the procedure should be discontinued, and short-acting nitrates along with oxygen should be administered immediately. 0.3–0.6 mg nitroglycerine tablet can be given sublingually and repeated every 5 minutes until the patient is relieved [48]. Emergency medical assistance must be summoned after three doses (in 15 minutes) if the anginal pain continues.

IHD patients are generally on antiplatelets or anticoagulants, and therefore care must be taken in case of excessive bleeding during the dental procedure by local hemostatic techniques. Discontinuation of these medications to perform minor surgical procedures is not recommended [49, 50]. If the patient is on anticoagulants, the International Normalized Ratio (INR) should be measured preferably within 24 hours before the procedure. A minor dental surgical procedure can be done safely with INR values in the range of 2.0–4.0 when local measures to control the bleeding are used [51, 52, 53, 54, 55, 56, 57]. If INR is more than 4.0, no surgical intervention should be performed without the cardiologist’s consultation.

## 4. Valvular Heart Diseases 

Valvular heart disease (VHD) is a cardiovascular disease caused due to damage or disease of heart valves. 2.5% of the US population has moderate to severe VHD and its prevalence increases with age [58]. Various forms of VHD include Degenerative valve disease, Rheumatic heart disease (RHD), and Congenital valve disease [59]. RHD is a common form of heart disease in developing nations while degenerative valve disease is more common in developed countries [60]. Common valvular disorders are aortic stenosis, aortic regurgitation, mitral stenosis, and mitral regurgitation. 2020 ACC/AHA Guidelines for the management of patients with VHD classified the progression of VHD into 4 stages (Table 1) [58].

**Table 1.** **Classification of Progression of VHD [58]**.

Stage	Definition
A	At-risk
B	Progressive
C	Asymptomatic severe
D	Symptomatic severe

Advanced valvular damage causes cardiac dysfunction leading to an increased risk of arrhythmia, infective endocarditis, stroke, and heart failure [61]. Due to the increased number of VHD patients visiting dental care centers, it has become imperative for dentists to have adequate knowledge of this disease for the safe and effective management of these patients. It is important to identify VHD before performing any dental procedure to avoid the risk of infective endocarditis (IE), risk of excessive bleeding due to anticoagulants, and worsening of the coexisting heart failure [62].

Dentists can play a vital role in the management of VHD and reducing the risk of complications like IE, stroke, and heart failure which can be fatal for affected patients [63, 64]. This can be done through regular dental checkups, patient education, and proper oral hygiene maintenance for these patients which minimize the risk of bacteremia caused by daily activities such as brushing and flossing [65]. In fact, maintaining good oral hygiene remains the most important element in preventing IE in VHD patients [58].

### 4.1 Prosthetic Valves

Most patients with severe VHD need intervention for the valve which includes valve repair or valve replacement (prosthetic valve) of the diseased valve. Prosthetic valves can be mechanical or biological (also called bio-prosthetic valves). Mechanical valves are more durable than bio-prosthetic valves but need lifelong anticoagulants such as warfarin and aspirin. Though all prosthetic valves come along with a risk of thromboembolism, bio-prosthetic valves are less thrombogenic than mechanical valves. The optimum INR range of anticoagulation for mitral mechanical valves is 2.5–3.5 while for aortic valves is 2–3 [66]. Thrombogenicity of older prosthetic valves is known to be higher and needs INR of up to 4 [66]. The main clinical implications for patients having prosthetic valves during dental treatment are (a) the need for antibiotic prophylaxis against infective endocarditis and (b) perioperative anticoagulation management [62].

Patients with prosthetic valves need proper dental care with appropriate antibiotic prophylaxis to prevent infective endocarditis. Patients with mechanical valves or high-risk patients with bio-prosthetic valves need appropriate anticoagulation with warfarin and aspirin. Anticoagulation in low-risk patients with bio-prosthetic valves can be managed by aspirin alone [67]. Most dental procedures involve minor bleeding which can be controlled easily and do not require cessation of anticoagulation therapy. They can be performed with an INR of up to 4.0. When anticoagulation cessation is necessary for patients with a high risk of thromboembolism like patients with the mechanical valve but no risk factors, warfarin can be stopped 48–72 hours prior to the procedure (so INR falls below 1.5) and reinitiated within 24 hours of the procedure after control of active bleeding [68, 69, 70]. In mechanical valve patients with the presence of risk factors, warfarin can be stopped 72 hours prior to the procedure and when INR falls below 2.0, heparin can be started which can be stopped 4–6 hours before the procedure [71]. Anticoagulants are restarted immediately after active bleeding control till therapeutic INR is reached again. Irrespective of the discontinuation of anticoagulation or not, INR must be recorded before any dental procedure involving significant bleeding [62]. The risk of reducing INR must be weighed against the risk of thromboembolism in consultation with the patient’s physician. Every attempt must be made to control the bleeding even after procedures like extraction or periodontal surgeries using local measures such as suturing, oxidized cellulose, gelatin sponge, thrombin, and tranexamic acid mouthwashes [62].

### 4.2 Infective Endocarditis

Infective endocarditis (IE) is an infection of the endocardial surface of the heart, which may involve one or more than one heart valve [72]. It can affect both native and prosthetic valves. The infection is usually seen in previously damaged heart valves or tissues. This disease has a low incidence but a high overall mortality rate. Thus, primary prevention and proper diagnosis are the main management strategies for this life-threatening disease. In industrialized countries, the estimated annual incidence of IE is 3 to 9 cases per 100,000 individuals [73, 74, 75, 76]. In-hospital mortality of this disease is 15–22% [77, 78] and the 5-year mortality rate is around 40% [79].

Infective endocarditis is predominantly a bacterial infection with 80% of the cases caused due to streptococci and staphylococci. Formerly, around 60% of native infective endocarditis cases were caused by streptococci. Now staphylococci are more commonly associated with several forms of IE [78, 80]. IE is more commonly observed among patients with prosthetic valves, mitral valve prolapse, congenital heart diseases, intracardiac devices, rheumatic heart disease, infective endocarditis history, or a history of cardiac valve surgery [81]. Prosthetic valve patients with Staphylococcus aureus IE have a high mortality rate of 40% or more [73].

The healthy valvular endothelium is resistant to microbial colonization but damaged valvular endothelium gets colonized by the circulating bacteria in the bloodstream forming infective vegetation and causing infection. The presence of valvular vegetation is a major criterion in the diagnosis of IE. Severe complications of IE include sepsis, paravalvular extension, embolic events, stroke, and heart failure. Management includes prolonged intravenous antibiotics and valvular surgery [82].

Various dental therapies and other invasive procedures have been commonly and controversially linked to implanting of bacteria in the heart valves leading to IE. Although IE does not pose an emergency in dental clinics, it is generally associated with significant morbidity and mortality. Any dental procedure that aggravates the bleeding and transfers oral bacteria to the bloodstream is an invasive dental procedure, for example scaling, periodontal therapy, exodontia, or any soft tissue surgery. The prevalence of transient bacteremia widely varies for different dental procedures: scaling and root planing (8%–80%), dental extraction (10%–100%), periodontal surgical procedure (36%–88%), and endodontic procedures (up to 20%) [83, 84, 85, 86, 87, 88, 89]. Even non-invasive dental procedures such as local anesthesia, band placement, and regular practices like tooth brushing and flossing (20–68%), toothpick use (20–40%), and chewing food (7–51%) can cause transient bacteremia [87, 88, 89, 90, 91, 92, 93]. Thus, the degree of cumulative bacteremia due to routine daily activities is far greater than the infrequent episodes of bacteremia caused by dental procedures.

Since 1955, several recommendations have been made by various expert committees and societies for antibiotic prophylaxis in preventing IE secondary to dental procedures [94]. These guidelines were based on the viewpoint that antibiotic prophylaxis can reduce or eliminate the bacteremia caused by dental or other invasive procedures and prevent IE. However, these recommendations were based on expert opinions and clinical experiences rather than double-blinded randomized controlled clinical trials. No controlled studies have revealed that antibiotic prophylaxis has decreased the incidence of IE. In fact, for many patients, the adverse effects of antibiotic prophylaxis outweigh its benefits. Injudicious antibiotic use can lead to the development of resistant organisms, drug toxicity, and excessive treatment cost [58].

A Cochrane Database systematic review in 2013 concluded that there is no evidence to ascertain that antibiotic prophylaxis is effective or ineffective in the prevention of IE following dental procedures [95]. In 2017 an extensive systematic review and meta-analysis was conducted by Cahill TJ *et al*. [96] which could not definitely conclude the effectiveness of antibiotic prophylaxis due to limited evidence. The lack of a strong evidence base in favor of antibiotic prophylaxis to prevent IE led to a major revision of AHA guidelines in 2007 wherein the writing committee recommended antibiotic prophylaxis only for the small subcategory of patients who are at the highest risk of developing IE [81]. UK National Institute for Health and Care Excellence (NICE) in 2008 also updated its guidelines and no longer recommended the use of antibiotic prophylaxis in the prevention of IE [97]. However, in 2016 NICE changed its guidelines which do not recommend antibiotic prophylaxis *routinely* for patients undergoing dental treatment [98]. According to this update, antibiotic prophylaxis may be appropriate for selected cases where the dentist perceives the risk of IE to be high or the patient prefers to have the prophylaxis.

According to the American Heart Association guidelines 2007, cardiac conditions which have the highest risk of adverse outcome from IE include those with prosthetic cardiac valve or prosthetic material used for cardiac valve repair, history of IE, congenital heart disease (CHD), and cardiac transplant recipients developing cardiac valvulopathy [81, 99]. For patients with the aforementioned cardiac conditions, antibiotic prophylaxis is rational for all dental procedures that involve gingival or periapical tissue manipulation or oral mucosal perforation. Prophylaxis is not required for dental procedures such as anesthetic injection through non-infected tissues, dental radiographs, suture removal, biopsies, placement or adjustment of orthodontic or removable prosthodontic appliances, and putting orthodontic brackets [81, 99].

Antibiotic prophylaxis is usually given in a single dose 30 to 60 minutes before the dental procedure but can be given up to 2 hours after the procedure. Amoxicillin is the drug of choice for adults (2 grams) and children (50 mg/kg) [81, 99]. For adult patients with amoxicillin allergy, azithromycin (500 mg)/clindamycin (600 mg)/clarithromycin (500 mg) or first or second-generation oral cephalosporin like cephalexin (2 grams) can be administered [81, 99]. Patients who are unable to take oral medications can be given ampicillin, ceftriaxone, or cefazolin intramuscularly (IM) or intravenously (IV). For patients who are allergic to ampicillin and cannot take oral medications - parenteral ceftriaxone, cefazolin, or clindamycin are recommended [81, 99]. The latest American Heart Association scientific statement (2021) recommends that in individuals with allergies to amoxicillin or ampicillin drugs, clindamycin must no longer be used as an oral or parenteral alternative as it may cause more frequent and severe drug reactions [99].

There is still a dearth of substantial scientific evidence to prove that dental procedures cause IE in patients with underlying cardiac conditions and antibiotic prophylaxis is worthwhile in preventing IE [81, 99]. Even 100% effective antibiotic prophylaxis can prevent only a small number of IE cases. The majority of the IE cases are caused due to bacteremia from routine daily activities including brushing, flossing, and chewing. There must be a fundamental change in the management of patients at high risk of IE by shifting the focus from antibiotic prophylaxis and dental procedures towards access to proper dental care and maintenance of optimal oral health.

## 5. Anticoagulants

Anticoagulant therapy or antithrombotic therapy has been used widely to manage various cardiovascular conditions including myocardial infarction, stroke, and deep vein thrombosis [100]. The patients on anticoagulant therapy should be handled with proper care and in consultation with the patient’s physician. The patients on anticoagulant medication and undergoing a dental procedure that involves low to medium risk of bleeding can be effectively managed by local hemostatic measures. AHA/ACC/ADA/ESC/SCAI/ACCP guidelines do not suggest the cessation of antiplatelet therapy for procedures involving a low risk of bleeding [101, 102, 103].

The ratio of the patient prothrombin time to the mean prothrombin time raised to the power of international sensitivity index (ISI) value is the INR of the patient. It determines the anticoagulation status of a patient [104].

$$I\,N\,R={\left(\frac{\text{ Patient }\,P\,T}{M\,e\,a\,n\,P\,T}\right)}^{I\,S\,I}$$

It is warranted to know the INR value of a patient on anticoagulation therapy before a dental procedure. It is recommended to check the INR 72 hours prior to an invasive dental procedure in a patient taking long-term anticoagulant therapy and stably anticoagulated on warfarin [105]. A physiologically normal patient has an INR value of 1, and a therapeutic range of 2.0 to 3.0 is considered safe for most indications due to the reduced risk of thromboembolic events [106]. In the case of prosthetic heart valves, a higher INR range of 2.5–3.5 is required [107]. Guidelines on the management of patients on oral anticoagulant therapy (2007) recommend not to discontinue the anticoagulants in patients with stable INR in the range of 2.0–4.0 as the risk of significant bleeding is low for most outpatient dental procedures [105]. American Academy of Oral Medicine (AAOM) Clinical Practice Statement 2016 stated that an INR value of 3.5 (up to 4.0 by some experts) is safe for moderately invasive dental surgical procedures like simple tooth extractions [108]. For non-invasive dental procedures, there is no need to maintain this safety margin [109].

If INR is above 3.5, then the dental procedures should be done with a medical professional consultation weighing the risk of thromboembolism [66]. In the case of novel non-VKA (non-vitamin K antagonists) and natural/direct anticoagulant therapy (NOAC/DOAC), no discontinuation of the anticoagulant is recommended for low bleeding risk procedures. These procedures can be effectively performed 18–24 hrs after the last dose and anticoagulant can be restarted after 6 hrs of treatment. The medication discontinuation during the medium-risk procedure should be in close communication with the patient’s physician [66, 103]. In cases of combined anticoagulant and antiplatelet therapy or triple therapy with 2 antiplatelets and one anticoagulant, consult the patient’s physician for individualized management [66]. The risk of thrombosis on temporary discontinuation of anticoagulants is small but life-threatening. Thus, the benefits of discontinuing anticoagulants must always be weighed against the risk of fatal thromboembolic events in consultation with the responsible medical professional.

### 5.1 Warfarin Therapy

**Fig. 1.**
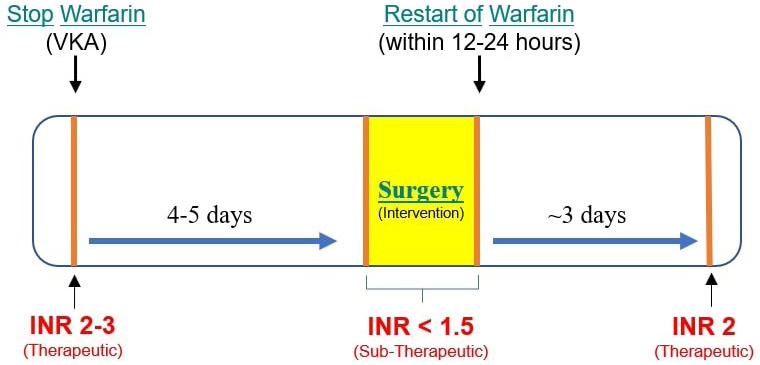
**Perioperative Warfarin therapy management**. Discontinuing warfarin for 4–5 days reduces the INR below 1.5 which is safe for dental surgical intervention. After restarting warfarin, it takes around 3 days for INR to reach a therapeutic level of 2 before which there is an increased risk of thromboembolism.

Warfarin, a vitamin K antagonist, is one of the most common oral anticoagulants used for the prevention and treatment of thromboembolism for more than 50 years. In terms of INR, warfarin has a therapeutic index of 2.0–3.0 [110]. Factors like narrow therapeutic range along with drug-drug and drug-food interactions limit its use. Over the years, dentists have practiced various perioperative bleeding management strategies for patients on anticoagulant therapy which ranged from continuing the regular dose of anticoagulant to reducing the dose to complete halting or using a bridging anticoagulant like heparin [111]. Dental procedures are generally categorized as low bleeding risk procedures and studies have concluded that anticoagulation therapy can be safely continued for most of these procedures including dental extractions [112]. The risk for embolic events after dental surgery appears to vary from 0.02 to 1%. According to a systematic review and meta-analysis (2009) while performing minor dental procedures, continuing the regular warfarin dose does not cause increased bleeding when compared with adjusting or discontinuing its dose [113]. A 2015 systematic review concluded that for patients undergoing dental extractions, a regular dose of warfarin can be continued if INR is within the therapeutic range [114]. The benefits of warfarin therapy discontinuation must be weighed carefully against the risk of thromboembolism. If deemed essential, warfarin must be discontinued five days before major surgery and restarted 12–24 hours postoperatively [115]. INR must be measured a day before surgery to monitor adequate reversal of anticoagulation [116]. When warfarin therapy is discontinued in patients with an initial INR of 2.0–3.0 for about 4 to 5 days, the INR falls to the normal range (less than 1.5) on the day of surgery which is considered safe for dental procedures and associated with acceptable risk of perioperative bleeding [117]. The INR reaches the therapeutic range (2.0) after around 3 days of restarting the warfarin therapy [116, 118, 119] (Fig. 1). These patients have a subtherapeutic INR for approximately 2–3 days preoperatively and 2 days after surgery during which they are at risk of a thromboembolic event [116, 120].

There have been reports of patients experiencing fatal or non-fatal thromboembolic events due to the stoppage of oral anticoagulation for dental treatment [56]. However, the majority of the patients had no severe effects. According to Todd *et al*. (2005) [121], an individualized approach to patients’ systemic conditions by acquiring proper history would be a rational approach in treating patients on warfarin therapy.

### 5.2 Direct Oral Anticoagulants (DOACS)

The classically used anticoagulant agents are the dicumarinic agents such as Warfarin (Coumadin®) and the acenocoumarol. The disadvantage of these agents is the need of dose adjustment for each patient, their interaction with the consumed foods, and a need to monitor the international normalized ratio (INR) regularly. To overcome these drawbacks, direct oral anticoagulants (DOACs) are being used nowadays [122]. DOACs are also known as New Oral Anticoagulants (NOACs) and common examples are apixaban, rivaroxaban, dabigatran etexilate, and edoxaban [122].

The newer agents do not require regular monitoring of anticoagulation and dose adjustment. Studies have also shown no need for NAOC therapy interruption for most dental treatments due to a low incidence of bleeding complications with NAOCs [123, 124]. However, the disadvantages of newer agents are higher cost, absence of proper reversal protocols, and lack of post-marketing clinical trials [125].

The risk of bleeding and adjustment of anticoagulant therapy with newer drugs depend on the type of dental procedure. Low-risk procedures have lesser chances of bleeding and include simple restorations, local anesthetic administration, supragingival scaling, and single-tooth extraction. Moderate risk procedures include extraction of 2 to 4 teeth and gingival surgery of up to 5 teeth. High-risk procedures are associated with a higher risk of bleeding and comprise extracting ≥6 teeth, generalized gingival surgery of ≥6 teeth, and multiple dental implant placement [126]. According to the available evidence, DOAC therapy can be continued safely for low-to-medium risk dental procedures as the involved risk of bleeding is low. In case bleeding occurs, local hemostatic measures can be used to manage it [125]. It is recommended not to perform these dental procedures during the peak concentrations of DOAC therapy but close to the end of the dosing cycle. Another alternative is to administer the DOAC after the procedure instead of before or perform the procedure 12–24 hours after the DOAC administration. In case of invasive or surgical dental procedure involving moderate to high risk of bleeding, it is recommended by manufacturers to discontinue edoxaban and rivaroxaban 24 hours prior and apixaban 48 hours before the procedure [127, 128, 129]. If discontinuation of DOAC is necessary, it should be reinitiated on the same day of the dental procedure. Overall, there is limited evidence at hand on the dental management of patients taking NAOCs and further studies are strongly recommended.

### 5.3 Bleeding Complications

The commonly used anticoagulants or the antiplatelets are the clopidogrel (Plavix®), ticlopidine (Ticlid®), prasugrel (Effient®), ticagrelor (Brilinta®), and/or asprin.The antiplatelet or anticoagulant therapy during the dental treatment increases the risk of bleeding which can be significant but generally not serious. Anticoagulants or antiplatelet drugs need not be stopped for procedures that induce minor bleeding, such as bridge placement, three teeth removal, and scaling procedures [54, 130]. If multiple extractions are planned, it has to be performed three teeth at a time in multiple visits choosing the most affected at the first visit [130, 131]. Most cases of perioperative or postoperative bleeding can be managed by minimizing the surgical trauma, limiting the area of surgery, primary closure of the surgical wound with sutures, applying pressure with gauze for 15–30 minutes, or using local hemostats such as tranexamic acid-soaked bite swabs, gelatin sponge, EACA (Epsilon amino caproic acid), 4.8% tranexamic acid mouthwash (4 times a day for 2 minutes, 1–2 days postsurgery), calcium sulfate (CaS), fibrin sponge, autologous fibrin glue, resorbable oxycellulose dressing and N-butyl-2-cyanoacrylate glue (NBCA) [132, 133, 134, 135]. The incidence of bleeding that local hemostatic methods could not manage ranges from 0 to 3.5% [57, 136].

Postoperative measures for the patients on oral anticoagulants to prevent bleeding include adequate resting, no vigorous rinsing or sucking; avoiding hot or hard foodstuffs that can disturb the socket with the tongue or any foreign object [137]. NSAIDs and COX-2 inhibitors should not be prescribed for analgesia to patients taking antithrombotic medications [135].

## 6. Arrhythmias

Cardiac arrhythmia or dysrhythmia can be described as a disturbance in the generation or conduction of cardiac electrical impulses which causes improper heart functioning [18]. Common heart rhythm abnormalities include premature atrial or ventricular beats, sinus bradycardia, and sinus tachycardia [138, 139]. The most common sustained cardiac arrhythmia found clinically is atrial fibrillation (AF) with approximately 46.3 million patients suffering from it globally [140]. Its incidence increases with obesity [140] and age, with 9% of the patients above 80 years having AF [141].

Dysrhythmia can be present in healthy people, patients on certain medications, with cardiovascular problems, or other systemic diseases. Many individuals with arrhythmia are asymptomatic, and the abnormality is generally found during a routine examination. However, others can present a variety of symptoms like dizziness, shortness of breath, weakness, syncope, and chest pain (angina). Elderly patients with compromised cardiac conditions can even develop myocardial ischemia, shock, or congestive heart failure due to arrhythmia [142, 143, 144, 145].

Patients with certain cardiac arrhythmias like atrial fibrillation have more chances of having ischemic events during stressful dental treatment or when excessive local anesthesia with a vasoconstrictor is administered. In addition, the association of arrhythmias with various systemic diseases and their potentially life-threatening nature make it imperative for dentists to identify and adopt appropriate measures to manage this cardiac problem promptly and effectively.

Arrhythmias are generally managed by antiarrhythmic drugs, cardiac pacemakers, cardioversion, or surgical intervention. Beta-blockers and calcium channel blockers are the most frequently prescribed medications. However, these drugs are associated with oral side effects like xerostomia and gingival overgrowth. These patients may seek attention from the dentist due to abnormal gingiva appearance (gingival enlargement), bleeding, pain in the gums, or dryness of mouth, and must be treated with caution. Cardiac pacemakers and automatic implantable cardioverter defibrillators (AICD) detect abnormal heart rhythm and help to restore the normal rhythm [146]. Cardioversion is the restoration of the sinus rhythm of the heart using medications or electric shock [147].

Identification of the patients with arrhythmia or those susceptible to developing arrhythmia, obtaining a detailed medical history, assessing vital signs, and consultation with the patient’s physician are vital steps to prevent the development of any severe complication during or after dental treatment. High-risk patients must be referred to a well-equipped hospital facility. In a dental office, stress and anxiety must be minimized to prevent the precipitation of arrhythmia. This can be achieved by premedication like short-acting benzodiazepines the night before or one hour before the appointment or by using nitrous oxide-oxygen inhalation.

Short morning dental appointments with a simple treatment plan are preferred. Local anesthetics with vasoconstrictors must be used judiciously as excessive epinephrine can precipitate arrhythmia or other unwanted cardiovascular complications [139, 148, 149]. The amount of vasoconstrictor should not exceed 0.04–0.054 mg per appointment [45]. In addition, vasoconstrictors like epinephrine are contraindicated in patients with refractory arrhythmias and must be used carefully in patients having implanted defibrillators or pacemakers [150].

There has been conflicting evidence in the literature regarding potential electromagnetic interference by electronic dental instruments like ultrasonic scalers, apex locators, and electrosurgical units in the functioning of cardiovascular implantable electronic devices (CIEDs) like pacemakers or implantable defibrillators. Many earlier in-vitro studies have found that dental electronic devices interfere with the functioning of CIEDs. Miller *et al*. [151] reported significant electromagnetic interference by electrosurgery units, ultrasonic bath cleaners, and ultrasonic scaling devices with pacemakers in a dental setup. Older pacemakers were unipolar and unshielded or poorly shielded. The newer ones are bipolar with better shielding which lowers the risk of malfunction due to electromagnetic interference [146].

Electric pulp testers, dental handpieces, amalgamators, curing lights, and sonic scalers have been reported to be safer [151]. Also, piezoelectric scalers are safer to use than magnetostrictive devices in patients with CIEDs [152, 153, 154]. Antibiotic coverage is not advocated by American Heart Association (AHA) for patients using devices like pacemakers and automatic defibrillators during invasive dental treatment [155]. Studies mention that an increased likelihood of electromagnetic interferences is seen if the device comes within 37.5 cm of the CIED or lead wire [151].

Arrhythmia patients may receive anticoagulant therapy, and therefore it becomes crucial for the dentist to determine the level of anticoagulation prior to any invasive procedure. Most dental procedures (including minor oral surgical procedures) can be performed if the International Normalized Ratio (INR) is 3.5 or less on the day of the procedure [156]. If the patient develops arrhythmia during dental treatment, the procedure should be deferred, and the patient must be urgently referred for medical evaluation. Oxygen should be provided, and the patient’s vital signs like body temperature, pulse, respiratory frequency, and blood pressure should be assessed. Loss of consciousness or collapse may indicate life-threatening arrhythmia or cardiac arrest, and emergency medical services must be activated immediately. Performing vagal maneuvers and placing the patient in Trendelenburg’s position can be helpful [146]. If no pulse is detected, cardiopulmonary resuscitation must be initiated using an automated external defibrillator without any delay.

## 7. Use of Local Anaesthetics with Vasoconstrictor

Dental treatment is often associated with pain, fear, and anxiety. Stress and anxiety may lead to exaggerated endogenous catecholamine release from the adrenal medulla, causing hemodynamic disturbances [157, 158]. In patients with underlying cardiovascular pathologies like hypertensive heart disease, ischemic heart disease, arrhythmias, or heart transplantation patients, inadequate local anesthesia may cause massive endogenous adrenaline release provoking cardiovascular complications. So, pain control and stress reduction are essential for patients undergoing dental treatment, particularly those with underlying cardiac disease.

Local anesthetics with vasoconstrictors (like epinephrine) in dentistry impart prolonged anesthesia, reduced systemic toxicity, and optimal bleeding control [159, 160]. Though the use of epinephrine in local anesthetics is common for healthy patients, its use is still debatable for cardiac patients due to its potential risk of causing unwanted cardiovascular effects. However, many authors have reported no clinically significant hemodynamic changes during dental treatment in healthy people or patients with mild to moderate coronary disease [22, 161, 162, 163, 164, 165].

The dose of vasoconstrictor used in dental treatment is significantly less than the dose used during the treatment of anaphylaxis for cardiac arrest. For example, one cartridge of local anesthetic of 1.8 mL in volume with 1:100,000 epinephrine contains only 0.018 mg of vasoconstrictor [166]. This small amount of vasoconstrictor used in dentistry poses little risk than the more significant risk from the massive endogenous epinephrine release due to improper control of pain and anxiety during dental treatment in cardiac patients. Thus, if administered carefully with aspiration, the concentration and amount of vasoconstrictor used in dentistry are generally not contraindicated for cardiac patients. However, it is better to use the lowest possible dose of vasoconstrictor to achieve adequate local anesthesia while treating patients with “stable” cardiac problems. Therefore, up to 0.04 mg or 40 μg of epinephrine, i.e., one or two carpules of 1.8 mL anesthetic solution with epinephrine 1:100,000 concentration is safe for controlled hypertensives and/or coronary disease patients [22, 167].

Heart conditions that contraindicate the use of vasoconstrictors in dentistry include [168]:

(a) Unstable angina

(b) Recent myocardial infarction

(c) Recent coronary artery bypass surgery

(d) Refractory arrhythmias

(e) Untreated or uncontrolled severe hypertension

(f) Untreated or uncontrolled congestive heart failure

Also, it is contraindicated to use epinephrine impregnated retraction cords, intraligamentary, and intrabony injections in these patients due to adverse hydrodynamic effects similar to I.V. epinephrine injection [168]. Though local anesthetics can lead to a decrease in the rate of amide metabolism in hypertensive patients taking beta-blockers [15], generally non-epinephrine-containing local anesthetics do not have significant drug interactions [169]. Drug interactions mostly stem from the incorporated vasoconstrictor [169]. For example, epinephrine can interact with commonly used antihypertensives like non-selective beta-blockers causing significant hypertensive episodes [170] and reflex bradycardia [15]. Though the response is dose-dependent and rarely seen in dental clinics, the dentist must be careful in using vasoconstrictors in patients with significant cardiac disease due to the serious sequelae of this drug combination. Agents like clonidine [171] and dexmedetomidine [172] can be used as safer alternatives to epinephrine with the local anesthetic solution in hypertensive patients. In such patients, it can also be prudent to use lidocaine, prilocaine, and mepivacaine solutions without vasoconstrictor [173].

## 8. Conclusions

Dental treatment, although considered safe, can be life-threatening if medical problems of the patient, especially cardiac disorders are ignored. A thorough medical and drug history from each patient at every appointment and extensive knowledge of the risk factors and clinical manifestations of various cardiac diseases can prevent many medical adversities in the dental clinic. A comprehensive treatment plan formulated in collaboration with the patient’s cardiologist can help avoid potential hazards during dental treatment for a cardiac patient. All drug prescriptions, surgical interventions, and overall management strategies should conform to the latest guidelines and protocols available.

Dentists should also identify and judiciously manage any medical emergency that can occur while the patient is on the dental chair. Stress before and during dental treatment is normal for any patient, but it can cause medical complications in cardiac patients. So pharmacologic agents can be prescribed to induce relaxation and reduce stress in these patients. Also, morning appointments should be preferred as mornings are more relaxing, and in case of any unforeseen situation, there is sufficient time to manage it.

Comprehensive knowledge of the dentist about cardiac conditions with multidisciplinary planning supported by an evidence-based approach is indispensable in imparting quality and safe dental care to this vulnerable cohort of dental patients.
